# Evidence for the Formation of Benzacridine Derivatives in Alkaline-Treated Sunflower Meal and Model Solutions

**DOI:** 10.3390/molecules21010091

**Published:** 2016-01-14

**Authors:** Verena Bongartz, Lisa Brandt, Mai Linh Gehrmann, Benno F. Zimmermann, Nadine Schulze-Kaysers, Andreas Schieber

**Affiliations:** 1 Department of Nutritional and Food Sciences—Chair of Molecular Food Technology, University of Bonn, Römerstraße 164, Bonn D-53117, Germany; vbongart@uni-bonn.de (V.B.); lisa.brandt89@gmx.de (L.B.); sakurabiyori@live.de (M.L.G.); benno.zimmermann@uni-bonn.de (B.F.Z.); schieber@uni-bonn.de (A.S.); 2 Institut Prof. Dr. Georg Kurz GmbH, Stöckheimer Weg 1, Köln D-50829, Germany

**Keywords:** amino acid, benzacridine derivatives, chlorogenic acid, green color, oxidation, *o*-quinone

## Abstract

Sunflower extraction meal (SEM) is an economically interesting protein source. During alkaline extraction of proteins, the presence of chlorogenic acid (CQA) in the meal gives rise to the formation of *o*-quinones. Reactions with nucleophiles present in proteins can lead to green discoloration. Although such reactions have been known for a long time, there is a lack of information on the chemical nature of the reaction products. SEM and model systems consisting of amino acids and CQA were subjected to alkaline treatment and, for comparison, to oxidation of CQA by polyphenoloxidase (PPO). Several green trihydroxy benzacridine (TBA) derivatives were tentatively identified in all samples by UHPLC-DAD-MS/MS. Surprisingly, in alkaline-treated samples of particular amino acids as well as in SEM, the same six TBA isomers were detected. In contrast, the enzymatically oxidized samples resulted in only three TBA derivatives. Contrary to previous findings, neither peptide nor amino acid residues were attached to the resultant benzacridine core. The results indicate that the formation of TBA derivatives is caused by the reaction between CQA quinones and free NH_2_ groups. Further research is necessary to elucidate the structure of the addition products for a comprehensive evaluation of food and feed safety aspects.

## 1. Introduction

Sunflower (*Helianthus annuus* L.) seeds are considered a promising source of proteins. Except for their low lysine content, sunflower proteins match the FAO (Food and Agriculture Organization) reference protein patterns in terms of amino acid composition and are low in antinutritive compounds. In addition to their relatively high nutritive value, sunflower proteins display various interesting functional characteristics comparable to those of soybean and other legume proteins, such as emulsifying or foaming properties [1]. The press residue originating after extraction of sunflower oils, in the following referred to as sunflower extraction meal (SEM), contains 40%–50% protein and is therefore an economically interesting source of nutrients [2].

SEM is also rich in phenolic compounds, with chlorogenic acid (CQA) being the predominant component. Depending on environmental and genetic factors, total phenolics may range from 1%–4% [1]. CQA has been associated with various health-promoting effects such as antioxidative, antibiotic or anti-inflammatory properties, which possibly prevent diseases associated with oxidative stress [3]. While highest protein yields are usually obtained by alkaline extraction, the presence of CQA in the meal gives rise to the formation of *o*-quinones under alkaline conditions. Additionally, *o*-quinones are formed in the presence of polyphenoloxidase (PPO) due to enzymatic oxidation. *o*-Quinones are electron-deficient molecules that readily react with nucleophiles, such as thiols or amino groups present in proteins. In intact plants, proteins and CQA are located in separate compartments, whereas in mechanically processed plants, CQA and proteins can interact with each other due to the ruptured cell structure. Such interactions have been shown to alter the physicochemical properties of proteins [4,5,6]. Furthermore, discoloration of SEM protein may occur, which limits its application in foods [2]. Although green discoloration as a result of the reaction of quinones and proteins has long been realized, only little information is available on the chemical nature of the reaction products. In model solutions containing caffeic acid esters, Namiki *et al.* detected green benzacridine derivatives, formed after reaction of quinones and lysine [7]. In the present study, SEM and model systems were subjected to alkaline treatment to investigate reactions between SEM protein and chlorogenic acid quinone. UHPLC-DAD-MS/MS analysis was used for the characterization of the resulting green adducts.

## 2. Results and Discussion

In the present study, SEM was subjected to alkaline treatment to investigate reactions between SEM protein and CQA quinone. Additionally, the 20 proteinogenic amino acids as well as NH_3_ were tested for their reactivity towards alkaline and enzymatically generated CQA quinone and to characterize the resulting adducts by LC/MS. It was expected that such a comparatively simple model system would allow conclusions to be drawn as to the situation in the intact protein.

### 2.1. Color Development in SEM Extract and Model Solutions

**Figure 1:**
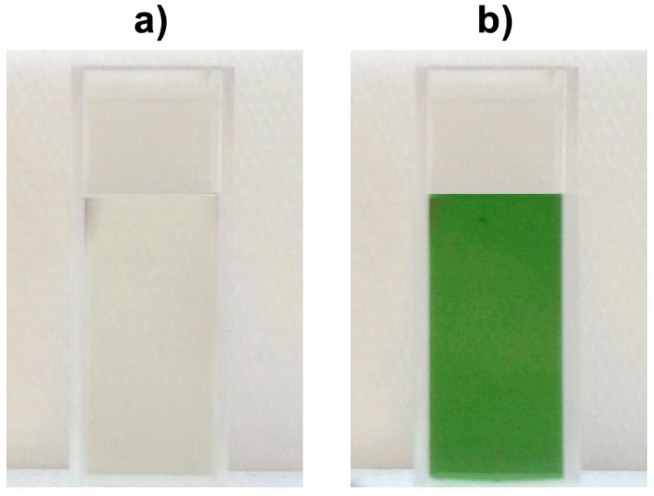
Extract of (**a**) non-alkalized SEM and (**b**) alkalized SEM (pH 9).

As shown in Figure 1, the non-alkalized SEM extract remained colorless (a), whereas alkalization of SEM extract resulted in green discoloration (b). Corresponding discolorations of sunflower meal and other plant materials such as sweet potato (*Ipomoea batatas*) or greater burdock (*Arctium lappa*) in alkaline surroundings are known to occur due to CQA quinone interactions with proteins [8,9]. In proteins, the ε-amino group of lysine and the thiol group of cysteine have been reported to be the preferred sites of reaction with quinones. Discoloration due to the addition of oxidized phenolic compounds to single amino acids such as tryptophan, histidine, and tyrosine has also been observed [10,11,12]. Namiki *et al.* and Yabuta *et al.* reported that, upon oxidation, CQA quinone gives rise to the formation of a dimer, which subsequently reacts with the amino compound to finally yield a green benzacridine derivative [7,9]. For the formation of such derivatives, Yabuta *et al.* concluded that an ortho-diphenol structure with a carbonyl side chain as present in CQA quinone is mandatory [9].

**Figure 2:**
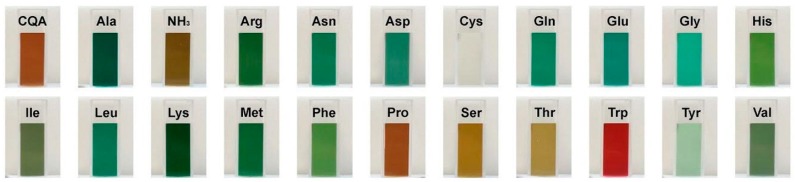
Color development in model systems containing CQA and individual amino acids at pH 9.

The incubation of CQA with the respective amino acid at pH 9 resulted in color formation including different shades of green, brown and, in the case of tryptophan, red (Figure 2).

All peaks showed absorption maxima at 631 and 461 nm. Thus, it can be assumed that the corresponding compounds are responsible for the coloration of the solutions. Since a green pigment had been observed upon reaction of primary amino groups with a dimer of oxidized ethyl caffeate [7,9], the green color may indicate attachment of CQA dimers to the α-NH_2_ group in all model systems, except for proline and cystein. Due to its highly nucleophilic character, cysteine reacted with CQA monomers instead of dimers and, consequently, did not form any colored adducts [11,12]. Among the tested amino acids, proline was the only amino acid with a secondary amino group. The reaction mixture with proline and CQA showed the same shade of brown as the CQA-solution without any added amino compounds. As indicated by Yabuta, a primary NH_2_ group is necessary for a reaction between a CQA-dimer and an amino acid [9]. Therefore, proline could not react with CQA to trihydroxy benzacridine (TBA). The red color in the alkaline reaction mixture with tryptophan and CQA can be explained by the presence of the indole group of tryptophan.

Contrary to alkalization, oxidation by PPO at pH 7 resulted in dark brown discoloration in all samples that resembled the brown color of oxidized CQA solution (see Figure 2). CQA quinones are known to readily polymerize, which leads to the formation of melanines. As incubation with PPO results in a large amount of *o*-quinones, it is reasonable to assume that the dark color is caused by melanines [7,13,14].

### 2.2. UHPLC-DAD-MS/MS Analysis

UHPLC-DAD-MS/MS analysis was used for the characterization of the resulting colored adducts. SIM (selected ion monitoring) was applied to detect specific molecule ions as derived from Prigent *et al.* [12] and Schilling *et al.* [15]. Positive ionization yielded higher intensities and was therefore used for further fragmentation experiments.

Although only 5-caffeoylquinic acid was used initially, incubation at 40 °C led to the formation of 3- and 4-caffeoylquinic acids that were identified by their characteristic fragmentation pattern as described by Clifford *et al.* [14]. With respect to the objectives of the present study, it was of greater interest that in all incubation mixtures a large number of peaks of low intensity were detected that corresponded to CQA dimers as described by Schilling *et al.* [15].

The CQA dimers are known to be the main compounds reacting with amino acids, as also observed for caffeic acid esters by Namiki and co-workers. It should be noted that several structures have been postulated for caffeic acid and CQA dimers, but only a few have been structurally elucidated [7,9,15].

**Figure 3:**
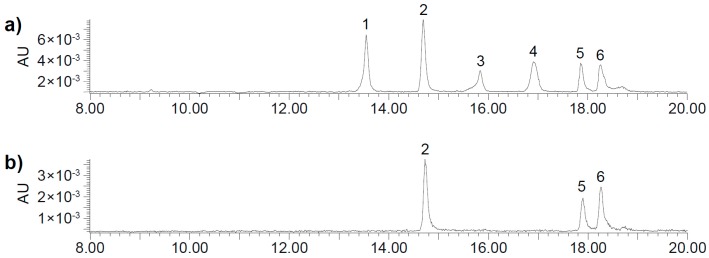
Exemplary UHPLC-DAD chromatograms of TBA isomers (450 nm) in (**a**) a model system containing α-alanine and CQA at pH 9 and in (**b**) a model solution containing α-alanine and PPO at pH 7.

**Figure 4:**
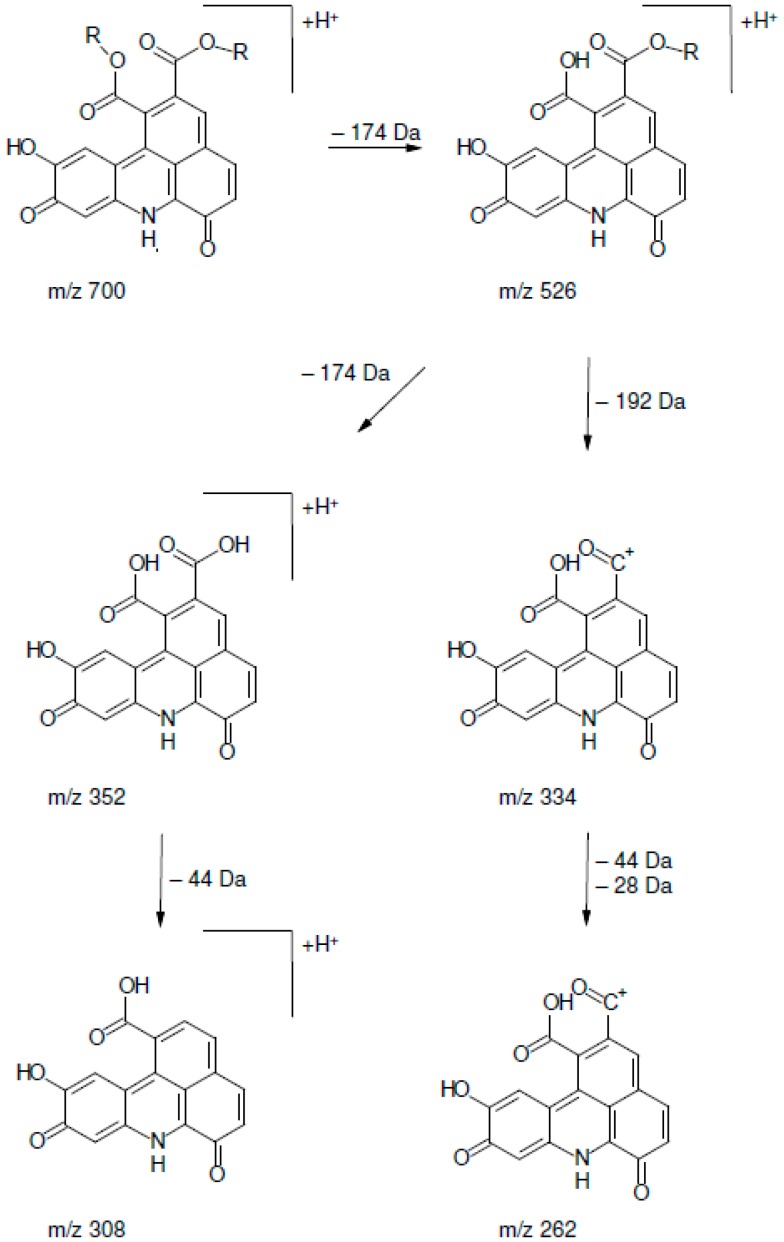
Postulated fragmentation pathway of a chlorogenic l-lysine adduct. R = Quinic acid Modified from [15]).

Although several distinct peaks were detected, in alkalized SEM extract and all alkaline model systems except for cysteine and proline, six peaks were dominant. Corresponding reaction mixtures containing PPO at pH 7 yielded three peaks that equaled three of the six peaks in the alkaline solutions (Figure 3). The mass spectrometric characteristics of the respective peaks are shown in Table 1 and Table 2. The latter findings are in agreement with Schilling *et al.* who detected three equivalent peaks in CQA solution containing *N*-BOC-lysine (*N*-butoxycarbonyl-lysine) and PPO. PPO is more specific and leads to fewer oxidation products than alkaline conditions. In alkaline solutions, 5-CQA isomerizes to 3-CQA and 4-CQA. Thus, the formation of CQA isomers is accounted for the occurrence of six peaks in alkaline solutions as opposed to three peaks in solutions containing PPO. All peaks displayed an *m*/*z* ratio of 700, matching fragments of corresponding reaction adducts as described by Schilling *et al.* The respective fragmentation pathway is given in Figure 4 [15].

**Table 1:** Mass spectrometric characteristics of trihydroxy benzacridine (TBA) isomers found in alkalized SEM extract. Model systems containing chlorogenic acid (CQA) and individual l-amino acids or NH_3_ at pH 9 showed identical peaks in terms of retention time and *m*/*z*. Corresponding model solution at pH 7 containing polyphenoloxidase (PPO) displayed only peaks 2, 5 and 6.

Peak	R_t_ (min)	[M + H]^+^	MS^2^ Fragments
1	13.51	700.3	334 (100), 352 (54), 526 (11), 262 (7), 308 (7), 654 (5) 700 (3)
2	14.65	700.3	334 (100), 352 (83), 526 (18), 262 (5), 308 (9), 654 (4), 700 (6)
3	15.80	700.3	334 (100), 352 (50), 526 (16), 262 (6), 308 (4), 654 (1), 700 (4)
4	16.87	700.3	334 (100), 352 (47), 526 (9), 262 (8), 308 (7), 654 (1), 700 (5)
5	17.79	700.3	334 (100), 352 (48), 526 (7), 262 (5), 308 (5), 654 (4) 700 (1)
6	18.20	700.3	334 (100), 352 (60), 526 (14), 262 (9), 308 (6), 654 (3), 700 (2)

**Table 2:** Mass spectrometric characteristics of additional TBA isomers found in the alkalized model system containing CQA and l-lysine. All peaks showed absorption maxima at 631 and 461 nm.

Peak	R_t_ (min)	[M + H]^+^	MS^2^ Fragments
1	13.48	829	334 (100), 128 (83), 352 (20), 526 (29), 336 (27), 654 (7) 700 (5), 130 (19), 318 (4), 480 (6)
2	14.59	829	334 (100), 128 (76), 352 (25), 526 (23), 336 (19), 654 (2) 700 (3), 130 (26) 318 (6), 480 (7)
3	15.73	829	334 (100), 128 (96), 352 (27), 526 (40), 336 (52), 654 (3), 700 (22), 130 (9), 318 (11), 480 (5)
4	16.74	829	334 (100), 128 (84), 352 (51), 526 (36), 336 (40), 654 (3), 700 (17), 130 (16), 318 (7), 480 (5)
5	17.73	829	334 (100), 128 (80), 352 (48), 526 (30), 336 (18), 654 (8), 700 (21), 130 (14), 318 (10), 480 (3)
6	18.11	829	334 (100), 128 (78), 352 (54), 526 (36), 336 (16), 654 (4), 700 (16), 130 (15), 318 (10), 480 (5)

**Figure 5:**
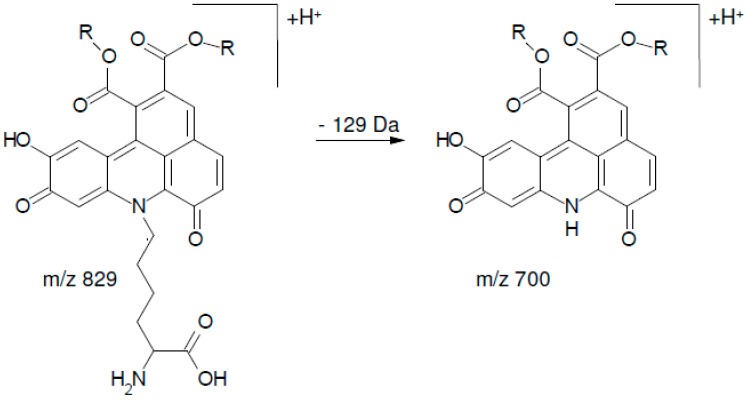
Postulated fragmentation of the chlorogenic l-lysine adduct with *m*/*z* 829 to the chlorogenic l-lysine adduct with *m*/*z* 700. R = Quinic acid. Modified from [15]).

Additionally, in the model solution containing lysine and PPO, three peaks with *m*/*z* 829 were observed that were dominant compared to the three peaks with *m*/*z* 700. Schilling *et al.* detected TBA derivatives with *m*/*z* 929 in a *N*-BOC-lysine solution, which fragmented to a molecule with *m*/*z* 700 due to the loss of the BOC-lysine residue (Δ 229). In this study, lysine, with a molecular mass of 146, was used instead of BOC-lysine, which also fragmented to a molecule with *m*/*z* 700 (Figure 5).

Therefore, the fragment at *m*/*z* 829 detected in the model solution containing lysine and PPO is explained by a TBA containing a lysine residue in agreement with the TBA-BOC-lysine adduct described by Schilling *et al.* [15]. The occurrence of peaks with *m*/*z* 700 in the same solution is explained by the additional NH_2_ group of lysine: the presence, or lack, of the lysine residue in the TBA molecule may depend on which NH_2_ group is incorporated in the TBA core. In all other model systems, exclusively peaks with *m*/*z* 700 were detected, *i.e.*, TBA cores without any side chains attached. All compounds showed a similar fragmentation pattern (Table 1 and Table 2), regardless if the [M + H]^+^ ion had *m*/*z* 700 or *m*/*z* 829. Although all peaks with *m*/*z* 700 were nearly identical in terms of fragmentation, they were detected at different retention times, suggesting that the corresponding compounds were structural isomers. These findings contradict Namiki *et al.*, who proposed a reaction mechanism for the formation of TBA derivatives with the corresponding amino acid side chain attached to the resulting benzacridine core [7]. It should be mentioned that Namiki *et al.* proposed the reaction mechanism on the base of *n*-butylamine, whereas Schilling *et al.* used *N*-BOC-lysine, thereby blocking the α-NH_2_ group of the amino acid. In this study, the α-NH_2_ group of all tested amino acids was available for the reaction as proposed by Namiki *et al.* for *n*-butylamine.

**Figure 6:**
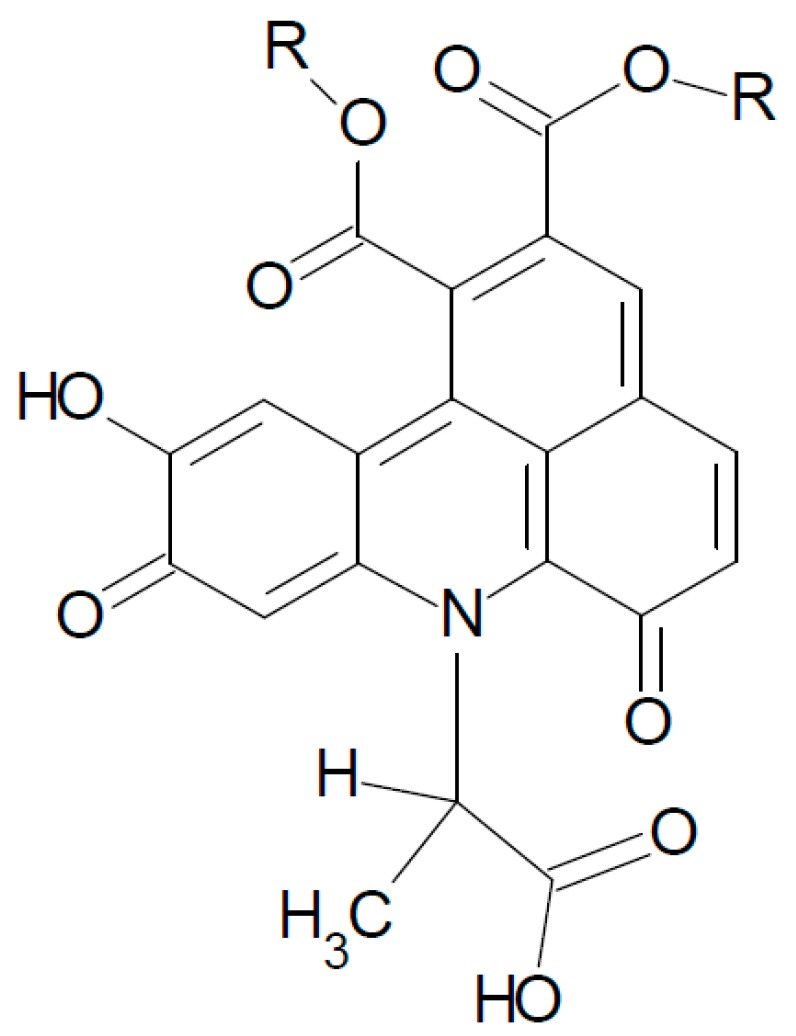
Postulated chlorogenic β-alanine adduct with *m*/*z* 772 in alkaline and enzymatically treated solutions. R = Quinic acid.

To verify that the reaction of the α-NH_2_ group is accountable for the loss of the amino acid side chain, β-alanine was used in additional CQA model systems. Neither alkaline nor enzymatic treatment resulted in peaks with *m*/*z* 700. Instead, in the enzymatically treated β-alanine solution, three peaks with *m*/*z* 772 were detected, accounting for TBAs with an alanine side chain attached (Figure 6). Furthermore, in the alkaline treated β-alanine solution, six peaks with *m*/*z* 772 were detected, which corresponded to the three or six peaks, respectively, shown in the respective α-alanine systems.

**Figure 7:**
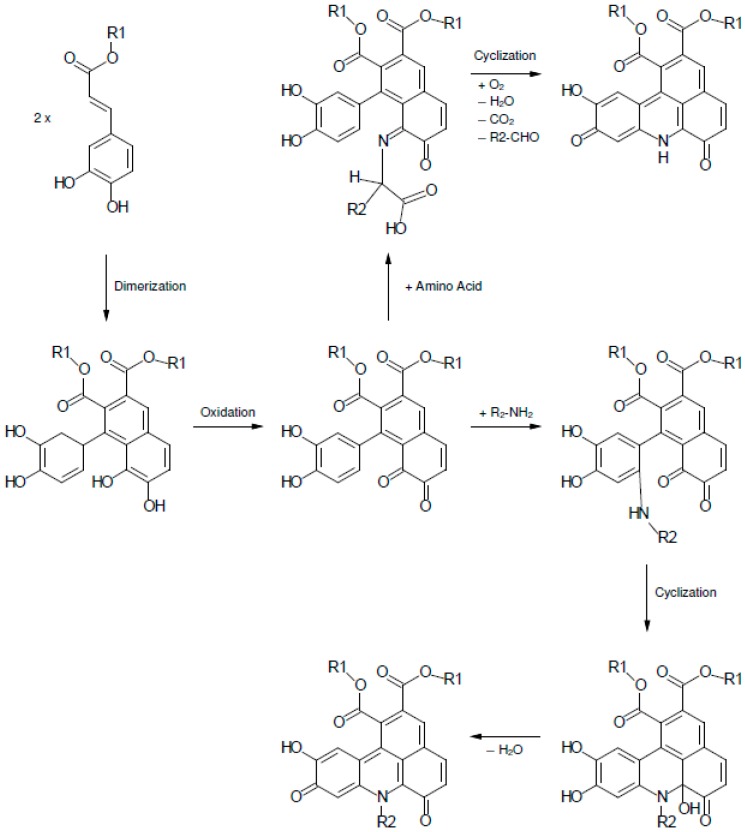
Proposed reaction mechanism between CQA and individual amino acids. If an α-NH_2_ group is involved, the resulting TBA core is free from any amino acid side chain. If TBA formation occurs at β- or ε-NH_2_ groups, corresponding amino acid side chains remain attached. R_1_ = Quinic acid. R_2_ = amino side chain.

These findings suggest that the presence, or lack, of an amino acid residue in the resulting TBA core depends on which NH_2_ group is involved in the TBA formation. If β- or ε-NH_2_ groups are incorporated in the TBA core, it can be assumed that the amino acid residue remains attached. This presumption is confirmed by recent research regarding milk and coffee proteins, in which ε-NH_2_ groups of lysine were found to be incorporated in proteins that had been modified with phenolics [16,17]. Generalizing the findings of Namiki and Schilling, a corresponding reaction mechanism is proposed in Figure 7, which postulates TBA formation as a result of interaction between CQA and α-amino acids or amino acids with β- or ε-NH_2_ groups [7,15].

## 3. Experimental Section

### 3.1. Plant Material

Sunflower extraction meal pellets were purchased from Cefetra B.V. (Rotterdam, The Netherlands). The pellets were ground using a model RETSCH ZM centrifugal mill (Haan, Germany) at 200 s^−1^.

### 3.2. Chemicals and Reagents

Solvents and reagents were purchased from VWR International (Darmstadt, Germany) and were of analytical, HPLC or MS grade. 5-*O*-Caffeoylquinic acid (5-CQA; >95%), amino acids (>98%) and mushroom tyrosinase (PPO) T3824 (1881 units/mg) were purchased from Roth (Karlsruhe, Germany), Rohm & Haas (Saint Priest, France) and Sigma-Aldrich (Steinheim, Germany), respectively. Deionized water was used throughout.

### 3.3. Sample Preparation—SEM Samples

#### 3.3.1. Alkaline Treatment of SEM

Alkaline treatment of the ground SEM was carried out in a beaker at 5 s^−1^ using a Stuart orbital shaker (Staffordshire, UK) for 90 min. SEM and water were mixed at a meal-to-water ratio of 0.125 g·mL^−1^. The suspension was adjusted to pH 9 with 1 M NaOH initially, and again after 10 and 30 min. To stop the reaction, the suspension was neutralized using 1 M HCl. The suspension obtained was dried for 12 h at 70 °C and ground again using the centrifugal mill at 200 s^−1^.

#### 3.3.2. Pressurized Liquid Extraction of SEM

Pressurized liquid extraction (PLE) was performed with a Dionex ASE 350 (Thermo Scientific, Idstein, Germany) system. For this purpose, 1 g SEM (before and after alkalization) were each mixed with diatomaceous earth (Thermo Scientific) and filled into 34 mL Dionex stainless steel cells. For extraction, the cell was filled with extraction solvent (water), pressurized (1500 psi), and heated (40 °C). Subsequently, the cell was rinsed with water (150% of the cell volume) and purged with a flow of nitrogen (100 psi, 60 s).

Aliquots of 1 mL were filtered through 0.2 μm Chromafil RC-20/15 MS filters (Macherey-Nagel, Düren, Germany) and immediately stored at −80 °C until analysis.

### 3.4. Sample Preparation—Model Systems with 5-CQA and Amino Acids or Ammonia

For alkaline oxidized model systems, 112 mM aqueous solutions of each of the 20 α-amino acids (see Figure 1) or NH_3_ or β-alanine and a 28 mM aqueous solution of CQA were mixed at a ratio of 1/1 (*v*/*v*) as reported by Prigent *et al.* [12]. The solutions were adjusted to pH 9 with 0.1 M NaOH initially, and again after 10 and 30 min. The samples were stirred for 24 h at room temperature. 28 mM solutions of CQA mixed with water (1/1, *v*/*v*) served as a control. To stop the reaction, the solutions were neutralized using 1 M HCl.

For enzymatic model systems, 28 mM aqueous solutions of each of the 20 amino acids or β-alanine or NH_3_ containing PPO (15 U·mL^−^^1^) and a 112 mM aqueous solution of CQA were mixed at a ratio of 1/1 (*v*/*v*) [12]. The solutions were adjusted to pH 7 with 0.1 M NaOH initially, and again after 10 and 30 min. The samples were stirred for 24 h at room temperature. To stop the reaction, the solutions were neutralized using 1 M HCl.

Aliquots of 1 mL were filtered through 0.2 μm Chromafil RC-20/15 MS filters (Macherey-Nagel) and immediately stored at −80° C until analysis.

### 3.5. UHPLC-DAD-MS/MS

An Acquity UPLC system from Waters (Milford, MA, USA) consisting of a binary pump (BSM), an autosampler (SM) cooled at 10 °C, a column oven (CM) set at 40 °C, a diode array detector (PDA) scanning from 190 to 500 nm, and an Acquity TQD triple-quadrupole mass spectrometer with an electrospray interface were used. The compounds were separated using an Acquity HSS-T3 RP18 column (150 mm × 2.1 mm; 1.8 μm from Waters with a guard column (5 mm × 2.1 mm). Eluent A was water/0.1% formic acid, and eluent B was acetonitrile/0.1% formic acid.

For the analysis of the green reaction products, the following gradient program was used: 0 min, 2% B; 20 min, 17.7% B; 20.5 min, 100% B; 22.5 min, 100% B; 23 min, 2% B; 25 min, 2% B. The flow rate was 0.4 mL/min. MS parameters were as follows: capillary voltage, 1.5 kV; cone voltage, 25 V; extractor voltage, 2.0 V; RF voltage, 0.1 V; source temperature, 150 °C; desolvation temperature, 450 °C; cone gas (nitrogen), 50 L/h; desolvation gas (nitrogen), 900 L/h. Monitoring was performed at 450 nm, representing the approximate absorption maxima of the resulting adducts, and the UV-Vis spectra were recorded from 190–700 nm (peak width 0.2 min). Negative ion mass spectra of the column eluate were recorded in the range *m*/*z* 500–2000. For the product ion scans, argon was used as the collision gas (0.22 mL/min).

## 4. Conclusions

We subjected SEM to alkaline treatment and obtained evidence of the formation of several green trihydroxy benzacridine derivatives using UHPLC-DAD-MS/MS analysis. For the first time, the formation of the same TBA isomers in model systems containing CQA and individual amino acids at pH 9.0 was demonstrated, regardless of the amino acid employed. During oxidation by polyphenoloxidase at pH 7.0, only three of these TBA isomers were found. Contrary to the formation of TBA derivatives reported by Namiki *et al.* and other groups, neither peptide nor amino acid residues were attached to the resultant benzacridine core, except for model systems containing β-alanine or lysine. Whether any amino acid residues remain attached to the TBA cores seems to depend on which NH_2_ group is involved in TBA formation. Taking together these results, the formation of TBA derivatives might be caused by the reaction between CQA quinones and free NH_2_ groups. Our findings show that food protein interactions with phenolic compounds are still poorly understood. With attention on polyphenolic compounds as potential food additives, further research needs to be dedicated to the underlying interaction mechanism and implications of these findings in food and feed quality and safety.
